# Are routine postoperative X-Rays justified in adolescent idiopathic scoliosis?

**DOI:** 10.1186/1748-7161-7-S1-O13

**Published:** 2012-01-27

**Authors:** A Vila-Casademunt, F Pellisé, M Domingo-Sàbat, J Bagó, A Matamalas, C Villanueva, E Cáceres

**Affiliations:** 1 Fundació Institut de Recerca Vall Hebron, Barcelona, Spain; 2 Hospital Vall Hebron, Barcelona, Spain

## Background

The clinical relevance of X-ray findings may not justify routine postoperative radiographic controls at 0, 3, 6, 12, and 24 months in adolescent idiopathic scoliosis (AIS) patients undergoing instrumented fusion with third-generation implants.

## Materials and methods

Full-spine X-rays and clinical records from the first 2 years’ postoperative follow-up in all AIS patients who underwent instrumented fusion in our center between 2005 and 2008 were independently analyzed by 2 investigators (consensus for discrepancies). The reviewers sought any clinical feature justifying X-ray control and any relevant radiologic finding [1].

## Results

Records from 43 patients (mean age 16.5 years, 93% women) were evaluated. A total of 414 (212 posteroanterior, 202 lateral) full-spine X-rays (9.6/patient) were performed during the first 2 postoperative years: 392 were available for analysis, and 391 had an associated clinical note. Excluding the 89 immediate postoperative films, only 48 of 325 (14.8%) were clinically justified: pain in 17 (34%) patients, clinical progression of deformity in 4 (8%) and previous X-ray finding in 29 (58%). All patients with clinical progression had a relevant X-ray finding. Pain was associated with a relevant finding in 23.5% of cases (positive predictive value 0.1); 7.4% of films with no clinical justification showed a relevant finding (negative predictive value 0.86). Only 4.3% of films led to a therapy change. Lower Risser sign increased the risk of having a relevant radiographic finding (p<0.05).

## Conclusions

Routine 3, 6, 12, and 24-month postoperative X-rays are not justified in AIS and should be avoided in mature, uncomplicated cases.
